# Draft Genome Sequence of *Roseovarius* sp. PS-C2, Isolated from Sekinchan Beach in Selangor, Malaysia

**DOI:** 10.1128/MRA.00673-21

**Published:** 2021-09-23

**Authors:** Nurfatini Radzlin, Suhaila Mohd Omar, Kok Jun Liew, Kian Mau Goh, Iffah Izzati Zakaria, Ummirul Mukminin Kahar

**Affiliations:** a Malaysia Genome Institute, National Institutes of Biotechnology Malaysia, Kajang, Selangor, Malaysia; b Department of Biochemistry, Faculty of Biotechnology and Biomolecular Sciences, Universiti Putra Malaysia, Serdang, Selangor, Malaysia; c Department of Biotechnology, Kulliyyah of Science, International Islamic University Malaysia, Kuantan, Pahang, Malaysia; d Research Unit for Bioinformatics and Computational Biology, Kulliyyah of Science, International Islamic University Malaysia, Kuantan, Pahang, Malaysia; e Faculty of Science, Universiti Teknologi Malaysia, Skudai, Johor, Malaysia; Loyola University Chicago

## Abstract

*Roseovarius* sp. PS-C2 is a bacterium that was isolated from Sekinchan Beach in Selangor, Malaysia, using an *ex situ* cultivation technique. Here, we present a high-quality annotated draft genome of strain PS-C2 and suggest potential applications of this bacterium.

## ANNOUNCEMENT

*Roseovarius* spp. are halophilic or halotolerant bacteria that thrive in various marine habitats (1). These bacteria have been reported as potential candidates for bioremediation and quorum sensing (2, 3).

*Roseovarius* sp. PS-C2 was isolated using a modified *ex situ* cultivation technique (4). Wet sediment and mud (0 to 15 cm from the top layer) were collected, using a sterilized laboratory scoop, from Sekinchan Beach in Selangor, Malaysia (3.5029N, 101.0945E). A sterilized corn cob was inserted into a 3-liter bioreactor filled with the collected sediment. After 2 weeks of incubation at 30°C and 80 rpm, samples were taken from the corn cob and spread onto marine agar (Condalab, Madrid, Spain). The PS-C2 strain was isolated, and genomic DNA was extracted using the Monarch genomic DNA purification kit (New England Biolabs, Ipswich, MA, USA), according to the manufacturer’s instructions. The 16S rRNA gene was amplified by PCR using 27F and 1492R primers (5), sequenced, and taxonomically identified using the NCBI BLASTn search and the EzBioCloud 16S database (6). The analyses revealed that PS-C2 showed greatest similarity (99.93%) to Roseovarius pacificus 81-2^T^ (GenBank accession number DQ120726.1). Here, we present the genome sequence and analysis of *Roseovarius* sp. PS-C2. The high-quality annotated draft genome of PS-C2 might provide insights into its potential biotechnological applications.

*Roseovarius* sp. PS-C2 was grown on marine agar (pH 6.5) at 30°C for 24 h. Subsequently, bacterial genomic DNA was extracted using the Monarch genomic DNA purification kit, according to the manufacturer’s protocol. A paired-end library was prepared using the standard protocol of the NEBNext Ultra DNA library preparation kit for Illumina (New England BioLabs). Sequencing was performed using the NovaSeq 6000 system with 150-bp paired-end reads (Illumina, San Diego, CA, USA). Sequence adaptors and low-quality reads were filtered using Trimmomatic v0.40 (7). *De novo* assembly of the genome was performed using SOAPdenovo v2.04 (8). The genome was analyzed and annotated using the NCBI Prokaryotic Genome Annotation Pipeline (PGAP) v5.20 (9). Genome comparison between strain PS-C2 and all 75 available genomes of *Roseovarius* spp. in the NCBI genome database (August 2021) was carried out using digital DNA-DNA hybridization (dDDH) via the Genome-to-Genome Distance Calculator (GGDC) v2.1 (10) and the average nucleotide identity (ANI) function in the EzBioCloud server (11). Carbohydrate-active enzymes (CAZymes) present in the genome of the PS-C2 strain were mined using dbCAN2 (12). Default parameters were used for all software tools, unless otherwise specified.

The sequencer generated 1,748,493,900 bases from 5,828,313 paired-end reads. The *Roseovarius* sp. PS-C2 genome showed coverage of 362×. The genome was assembled into 39 contigs, with a total size of 3,958,657 bp, an *N*_50_ value of 411,343 bp, and a G+C content of 62.1%. The PS-C2 genome consisted of 3,920 predicted genes, of which 3,839 were protein-coding sequences, 31 were pseudogenes, 3 were rRNAs, 44 were tRNAs, and 3 were small RNAs. The genome comparison analyses showed that PS-C2 was closely related to Roseovarius pacificus 81-2^T^ (dDDH, 60.7%; ANI, 95.0%). Because the dDDH value was <70% (13) and the ANI value was <96% (11), the results indicated that strain PS-C2 is likely a new species of *Roseovarius.* The PS-C2 genome encoded 56 CAZymes, including 13 glycoside hydrolases (GHs), 31 glycosyl transferases, 8 auxiliary activity enzymes, and 4 carbohydrate esterases. Among the GHs, 4 lytic transglycosylases (LTs) belonged to GH family 103. LTs play an essential role in bacterial cell wall metabolism and present an attractive new target for antibiotic development (14). To date, none of the *Roseovarius* LTs has been characterized. Collectively, the PS-C2 genome contributed to the body of knowledge and possible applications of *Roseovarius* spp.

### Data availability.

The whole-genome shotgun sequence of *Roseovarius* sp. PS-C2 has been deposited in NCBI GenBank under BioProject accession number PRJNA716475, BioSample accession number SAMN18388700, and GenBank accession number JAHKSQ000000000. The version described in this paper is the first version, JAHKSQ00000000.1. The raw sequencing reads have been deposited in the NCBI Sequence Read Archive (SRA) with accession number SRR15311899. The 16S rRNA gene sequence of *Roseovarius* sp. PS-C2 has been deposited in NCBI GenBank with accession number MW785753.1.

## References

[1] Lu L, Zhang Y, Peng X, Liu J, Qin K, Peng F. 2020. *Roseovarius arcticus* sp. nov., a bacterium isolated from arctic marine sediment. Int J Syst Evol Microbiol 70:2072–2078. doi:10.1099/ijsem.0.004018. 32118527

[2] Bruns H, Ziesche L, Taniwal NK, Wolter L, Brinkhoff T, Herrmann J, Müller R, Schulz S. 2018. *N*-Acylated amino acid methyl esters from marine *Roseobacter* group bacteria. Beilstein J Org Chem 14:2964–2973. doi:10.3762/bjoc.14.276. 30591820 PMC6296433

[3] Chernikova TN, Bargiela R, Toshchakov SV, Shivaraman V, Lunev EA, Yakimov MM, Thomas DN, Golyshin PN. 2020. Hydrocarbon-degrading bacteria *Alcanivorax* and *Marinobacter* associated with microalgae *Pavlova lutheri* and *Nannochloropsis oculata*. Front Microbiol 11:572931. doi:10.3389/fmicb.2020.572931. 33193176 PMC7655873

[4] Chaudhary DK, Khulan A, Kim J. 2019. Development of a novel cultivation technique for uncultured soil bacteria. Sci Rep 9:6666. doi:10.1038/s41598-019-43182-x. 31040339 PMC6491550

[5] Chen YL, Lee CC, Lin YL, Yin KM, Ho CL, Liu T. 2015. Obtaining long 16S rDNA sequences using multiple primers and its application on dioxin-containing samples. BMC Bioinformatics 16(Suppl 18):S13. doi:10.1186/1471-2105-16-S18-S13. PMC4682383 26681335

[6] Yoon SH, Ha SM, Kwon S, Lim J, Kim Y, Seo H, Chun J. 2017. Introducing EzBioCloud: a taxonomically united database of 16S rRNA gene sequences and whole-genome assemblies. Int J Syst Evol Microbiol 67:1613–1617. doi:10.1099/ijsem.0.001755. 28005526 PMC5563544

[7] Bolger AM, Lohse M, Usadel B. 2014. Trimmomatic: a flexible trimmer for Illumina sequence data. Bioinformatics 30:2114–2120. doi:10.1093/bioinformatics/btu170. 24695404 PMC4103590

[8] Luo R, Liu B, Xie Y, Li Z, Huang W, Yuan J, He G, Chen Y, Pan Q, Liu Y, Tang J, Wu G, Zhang H, Shi Y, Liu Y, Yu C, Wang B, Lu Y, Han C, Cheung DW, Yiu SM, Peng S, Xiaoqian Z, Liu G, Liao X, Li Y, Yang H, Wang J, Lam TW, Wang J. 2015. SOAPdenovo2: an empirically improved memory-efficient short-read de novo assembler. GigaScience 4:30. doi:10.1186/s13742-015-0069-2. 26161257 PMC4496933

[9] Tatusova T, Dicuccio M, Badretdin A, Chetvernin V, Nawrocki EP, Zaslavsky L, Lomsadze A, Pruitt KD, Borodovsky M, Ostell J. 2016. NCBI Prokaryotic Genome Annotation Pipeline. Nucleic Acids Res 44:6614–6624. doi:10.1093/nar/gkw569. 27342282 PMC5001611

[10] Auch AF, Von Jan M, Klenk H, Göker M. 2010. Digital DNA-DNA hybridization for microbial species delineation by means of genome-to-genome sequence comparison. Stand Genomic Sci 2:117–134. doi:10.4056/sigs.531120. 21304684 PMC3035253

[11] Yoon SH, Ha S, Lim J, Kwon S, Chun J. 2017. A large-scale evaluation of algorithms to calculate average nucleotide identity. Antonie Van Leeuwenhoek 110:1281–1286. doi:10.1007/s10482-017-0844-4. 28204908

[12] Zhang H, Yohe T, Huang L, Entwistle S, Wu P, Yang Z, Busk PK, Xu Y, Yin Y. 2018. dbCAN2: a meta server for automated carbohydrate-active enzyme annotation. Nucleic Acids Res 46:W95–W101. doi:10.1093/nar/gky418. 29771380 PMC6031026

[13] Meier-Kolthoff JP, Klenk HP, Göker M. 2014. Taxonomic use of DNA G+C content and DNA-DNA hybridization in the genomic age. Int J Syst Evol Microbiol 64:352–356. doi:10.1099/ijs.0.056994-0. 24505073

[14] Dik DA, Marous DR, Fisher JF, Mobashery S. 2017. Lytic transglycosylases: concinnity in concision of the bacterial cell wall. Crit Rev Biochem Mol Biol 52:503–542. doi:10.1080/10409238.2017.1337705. 28644060 PMC6102726

